# Developing BET-ter treatments for cutaneous T-cell lymphoma

**DOI:** 10.18632/oncotarget.26662

**Published:** 2019-02-12

**Authors:** Henry K. Wong

**Affiliations:** Henry K. Wong: Department of Dermatology, University of Arkansas for Medical Sciences, Little Rock, AR, USA

**Keywords:** lymphoma, T cell, bromodomain, mycosis fungoides, sezary syndrome

Mycosis Fungoides (MF) and Sezary Syndrome (SS) are the most common subtypes of cutaneous T-cell lymphoma (CTCL), a heterogeneous group of extranodal non-Hodgkin’s lymphomas (NHL) of the skin that includes the CD30-positive lymphoproliferative disorders [1]. The tumor cell in MF and SS most often exhibit a phenotype of a mature, skin-homing, effector memory T-cell with immune markers positive for CD45RO+, CD4+, CLA+ and CCR4+. While patients in the early stages with patches and plaques have a generally indolent course, a subset of CTCL patients progresses and develops more extensive skin and organ involvement that requires systemic treatment [2]. In these patients with more advance clinical stages, there is a worse prognosis with markedly reduced 5 year survival [2].

Although many treatment options are available for CTCL, a cure does not exist with our current therapeutic repertoire [3]. There are numerous therapies available for early MF that are clinically effective and patients can receive sustain treatments over long durations to delay or prevent progression [4]. Occasionally patients may develop long-term remission. Despite having these effective treatments, relapse can occur and worse yet, these patients progress to more advanced stages with reater tumor burden. Unfortunately, the number of available therapies for these more affected patients are limited, and achieving durable efficacy remains a challenge. While the number of therapies are increasing, there remains a need for innovative therapy or combinations to improve response and extend durability in advance stages of CTCL. Thus there remains a significant unmet need for development of novel effective therapies in the treatment of refractory CTCL.

Recently, genetic approaches have been taken to shed insight into the molecular defects in CTCL and to highlight pathways that play a role in oncogenesis. From a number of high resolution sequencing studies of CTCL genomes, single nucleotide analysis has identified genes and potential pathways that are abnormal in CTCL [5, 6]. A frequent finding is mutation affecting the *MYC* oncogene in up to 42% of leukemic CTCL patients, highlighting the importance of this pathway as a potential target in treatment [5].

With the rapid advances in development of small molecules from combinatorial chemistry and genomic identification of pathways that are important in cell proliferation that play a role in cancer, novel compounds have been designed that can interact with specific targets to affect pathways important in cancer [7, 8]. A first step prior to controlled clinical trials is to validate that these compounds have activity *in vitro* in appropriate cancer cells.

Bromodomain and extra-terminal domain (BET) family of epigenetic regulatory proteins play an important role in cancer and one target for regulation is the proto-oncogene *MYC* which is abnormal in CTCL [9]. BET proteins recognize and bind to acetylated lysine residues of histone proteins, and modulate transcription of *MYC* and *BCL2* genes. Inhibitors of BET proteins have been shown to be active in hematologic and non-hematologic malignancies. In an innovative study by Kim *et al.*, the authors explore targeting the MYC pathway with BET inhibition, with the goal that inhibition of BET proteins disrupt transcription of *MYC* and alter growth of CTCL cells. Kim *et al.* showed JQ-1, a BET inhibitor, possess activity in reducing viability using cell lines derived from CTCL. Additionally and more importantly, they demonstrated inhibitory activity in primary neoplastic cells isolated from a cohort of CTCL patients, both MF and SS. The inhibitory response was observed at the micromolar level and affected patient derived neoplastic T cells from CTCL patients with significant blood involvement with similar sensitivity and independent of *MYC* amplification. The activity is not isolated to one BET inhibitor alone, but a number of different BET inhibitors, and supports the importance of the BET pathway in survival of CTCL T cells. Thus targeting this regulatory pathway offer promise in further clinical studies. The observed activity of multiple BET inhibitors in this preliminary study alsooffers potentially better future BET inhibitors that can be optimize for maximal toxicity to neoplastic cells, yet have minimal effect on normal cells. The unique findings open promising approach with BET inhibitor to treating CTCL.

As multiple pathways have been identified to be abnormal in cancer, one strategy to enhance efficacy in the treatment of cancer is to use multiple drugs to target differnet pathways, with the goal to increase effectiveness by synergism. Applying this approach of combination chemotherapy, the authors combined BET inhibitors with HDACs to study synergism between agents that individually have activity and are FDA approved in the treatment of CTCL. The results from using BET inhibitors with HDAC inhibitor, vorinostat or romedepsin, demonstrated that there was significant synergism observed in the reduction of viability of patient derived cells with multiple BET inhibitors. The combination treatments of BET inhibitor and HDAC inhibitor induced cells to undergone apoptosis with greater inhibition of *MYC* and *BCL2* expression in patient derived cells. The level of inhibition was significantly greater than either BET-inhibitor or HDAC inhibitor alone, 15-fold decrease with vorinostat and 80-fold decrease with romedepsin. A similar observation was seen with combining BET inhibitor with venetoclax, a BCL2 inhibitor, suggesting BET inhibitors can synergize with multiple agents.

In summary, these promising *in vitro* studies highlight the potential application of novel combinations of BET inhibitor and HDAC inhibitor or BCL2 inhibitor for the treatment of CTCL. The encouraging results suggest that these BET inhibitor combinations can be translated from genetic discoveries into testable and actionable combination strategies to meaningful clinical trials.
